# Pan-Cancer Analysis Identifies LUC7L2 as a Prognostic Biomarker and Potential Functional Regulator of Prostate Cancer Progression

**DOI:** 10.3390/biomedicines14102294

**Published:** 2026-10-09

**Authors:** Jialin Yuan, Ping Zhan, Jun Liu, Guangming Lu, Han Wang, Meiqin Xiao, Dandan Li

**Affiliations:** 1 Department of Oncology, Dongguan Hospital of Guangzhou University of Chinese Medicine, Dongguan 523000, China; 13580702248@163.com (J.Y.); wanghan11031103@sina.com (H.W.); 2 Department of Radiation Oncology, Dongguan Hospital of Guangzhou University of Chinese Medicine, Dongguan 523000, China; xmeiqin1989@163.com; 3 Department of Surgery, Dongguan Hospital of Guangzhou University of Chinese Medicine, Dongguan 523000, China; adde_edin@163.com (P.Z.); gddglgm@sina.com (G.L.); 4 Department of Laboratory Medicine, Dongguan Hospital of Guangzhou University of Chinese Medicine, Dongguan 523000, China; liuyu8566@126.com

**Keywords:** LUC7L2, prostate cancer, biomarker, multi-omics, DNA damage

## Abstract

**Background/Objectives:** Cancer imposes a substantial global public health burden, and its initiation and progression involve complex dysregulation of gene expression regulatory networks. **Methods:** This study systematically evaluated the expression characteristics and prognostic value of LUC7L2 across pan-cancer types, with a particular focus on functional validation in prostate cancer. Using transcriptomic and proteomic data from multiple databases, immunohistochemical staining from the Human Protein Atlas, and single-cell transcriptomic datasets GSE137829 and GSE176031, we analyzed the expression patterns of LUC7L2 in various cancer types. Diagnostic and prognostic potential was assessed through ROC curve analysis, Cox regression, single-cell, and spatial transcriptomics, while the functional role of LUC7L2 in prostate cancer tumorigenesis and progression was validated via in vitro assays. **Results:** Results showed that LUC7L2 was significantly upregulated in glioblastoma and prostate adenocarcinoma, and downregulated in lung adenocarcinoma and ovarian serous cystadenocarcinoma, with diagnostic AUC values exceeding 0.7 across multiple tumor types. In prostate cancer, high LUC7L2 expression was significantly associated with a shortened progression-free interval (multivariate HR = 3.50, 95% CI 1.95–6.29, *p* < 0.001), with a meta-analysis of independent cohorts showing a consistent direction of effect (pooled HR = 1.14, 95% CI 0.90–1.44). Moreover, LUC7L2 exhibited strong correlations with microsatellite instability and tumor mutational burden across multiple cancer types. Single-cell analysis revealed that its expression was markedly higher in malignant cells than in immune or stromal cells. In vitro knockdown of LUC7L2 in prostate cancer cells led to significantly suppressed proliferation, increased apoptosis, S-phase cell cycle arrest, reduced invasion and migration capacities, enhanced senescence, and aggravated DNA damage. **Conclusions:** This study demonstrates that LUC7L2 is specifically overexpressed in malignant prostate cancer cells and independently associated with poor prognosis. It further validates its regulatory roles in proliferation, apoptosis, migration, and DNA damage repair, providing both theoretical and experimental evidence supporting LUC7L2 as a potential biomarker for prostate cancer.

## 1. Introduction

Prostate cancer (PC), the most common histologic form of which is prostate adenocarcinoma (PRAD, the TCGA designation), remains one of the most prevalent malignancies in men worldwide, posing a substantial clinical challenge due to its high recurrence rate and metastatic potential [1]. Despite the widespread use of prostate-specific antigen (PSA) screening, imaging, surgical resection, radiotherapy, and androgen deprivation therapy, limitations persist, including insufficient specificity for early diagnosis, suboptimal prognostic accuracy, and a lack of targeted strategies for recurrent or treatment-resistant disease [2]. Prognosis currently relies largely on Gleason score and TNM staging, but existing biomarkers are insufficient for reliably predicting recurrence risk. Although several molecules—such as UBE2C, MAL2, SLC45A2, and TREM2—have been linked to PRAD progression and poor outcomes, many key regulatory factors remain unidentified [3,4,5]. Moreover, oxidative stress and the accompanying DNA damage response are increasingly recognized as important contributors to prostate carcinogenesis and progression, promoting genomic instability and therapy resistance [6]. ROS also promote lipid peroxidation and protein oxidation, and activate oncogenic signaling pathways such as NF-κB and PI3K/Akt, thereby fostering a pro-tumorigenic microenvironment [7]. In addition, chronic inflammation and androgen receptor signaling interact with oxidative stress to drive prostate cancer initiation and progression [8]. Recent integrative bioinformatics and machine-learning studies have further identified novel molecular biomarkers and multi-gene models for prostate adenocarcinoma [9,10]. The discovery of novel biomarkers and therapeutic targets is therefore essential for improving the clinical management of PRAD.

LUC7L2, a U1 snRNP-associated splicing factor, has been increasingly implicated in tumorigenesis. In myeloid neoplasms, deletions in the 7q34 region—where LUC7L2 resides—are considered important oncogenic events, and its mutation or deletion leads to haploinsufficiency [11,12]. Functional studies have demonstrated that LUC7L2 not only participates in 5′ splice site recognition and selection, but also reshapes cellular energy metabolism through alternative splicing; its loss enhances oxidative phosphorylation while suppressing glycolysis [13,14,15]. Additionally, LUC7L2 has been shown to negatively regulate innate immune responses by mediating MITA/STING intron retention and to contribute to radioresistance in nasopharyngeal carcinoma via the modulation of SQSTM1 expression [16,17]. In hepatocellular carcinoma, LUC7L2 promotes proliferation by enhancing RRAS-dependent DNA damage repair [18], while in glioblastoma, it facilitates temozolomide resistance through H3K9 lactylation-mediated MLH1 intron retention [19]. Moreover, CRISPR screens have identified LUC7L2 as a potential dependency gene in chordoma and glioblastoma. However, despite these insights in individual cancer types, a systematic integration of LUC7L2 expression across pan-cancer contexts, its relationship with genomic stability and epigenetic regulation, and its independent prognostic value and functional mechanisms in PRAD remain lacking.

As a member of the splicing factor family, LUC7L2 is likely involved in RNA processing and gene expression regulation across multiple cancers. Yet its precise expression pattern, prognostic relevance, and biological functions in PRAD have not been validated at the transcriptomic and proteomic levels. While single-cell and spatial transcriptomic technologies have provided new perspectives on tumor heterogeneity, the specific expression of LUC7L2 in malignant PRAD cells and its spatial microenvironmental context remain poorly defined. More critically, in vitro functional studies are essential to establish whether LUC7L2 acts as a promoter or suppressor of PRAD progression, but such evidence is still extremely limited, hindering its further clinical translation as a potential prognostic marker and therapeutic target.

To address these gaps, we integrated multiple public databases—including TCGA, GTEx, CPTAC, and HPA—to systematically construct a comprehensive landscape of LUC7L2 mRNA and protein expression across diverse cancer types and evaluate its diagnostic performance. Through the combined analysis of genomic instability markers and methylation profiles, we uncovered potential regulatory relationships between LUC7L2 expression and microsatellite instability, tumor mutational burden, and promoter methylation. Pan-cancer prognostic assessment and meta-analysis of independent PRAD cohorts further identified LUC7L2 as an independent predictor of progression-free interval (recurrence-free survival) in prostate adenocarcinoma. Single-cell and spatial transcriptomic data further revealed its elevated expression in malignant cells and defined its spatial localization pattern. Complementarily, pathway enrichment analysis, transcription factor interaction prediction, and in vitro functional knockdown experiments systematically validated the regulatory roles of LUC7L2 in prostate cancer cell proliferation, apoptosis, cell cycle progression, migration, senescence, and DNA damage repair. These findings provide a novel molecular target and mechanistic insights for precision diagnosis and the treatment of PRAD.

## 2. Materials and Methods

### 2.1. Data Acquisition and Processing

Gene expression data for LUC7L2 across 29 cancer types were obtained from The Cancer Genome Atlas (TCGA) database. To expand the control sample size, TPM values from normal tissue samples in the Genotype-Tissue Expression (GTEx) project were integrated. Batch effects introduced by different platforms were corrected using the ComBat algorithm implemented in the SVA package. Quantitative protein expression data for LUC7L2 were retrieved from the Clinical Proteomic Tumor Analysis Consortium (CPTAC); only cases with paired tumor and adjacent normal tissues were included. Protein expression values were Z-score normalized to eliminate systematic inter-sample variation. Immunohistochemistry staining results for LUC7L2 protein across 20 tumor types were obtained from the Human Protein Atlas (HPA) database. Using tumor versus normal tissue as the binary outcome and LUC7L2 expression level as the predictor, receiver operating characteristic (ROC) curves were generated for each cancer type with the pROC package to evaluate discriminatory ability.

### 2.2. Correlation Analysis Between Genomic Instability and LUC7L2 Expression

Twelve genomic instability indicators were selected, including copy number variation burden (FGA, FGG, FGL), homologous recombination deficiency-related scores (HRD, TAI, LST, LOH), aneuploidy score, tumor mutational burden (non-synonymous TMB and mutation count), and microsatellite instability scores (MSI MANTIS, MSI SENSOR). For each cancer type, Spearman’s rank correlation coefficients between these indicators and LUC7L2 expression were calculated using R’s (version 4.3.2) cor.test function (method = “spearman”). Only cancer types with paired effective sample sizes ≥ 10 were included in the statistical analysis. *p* values from these pan-cancer correlation analyses were adjusted for multiple testing using the Benjamini–Hochberg false discovery rate (FDR) method, and associations with FDR < 0.05 were considered significant.

### 2.3. Survival Prognosis Analysis

To assess the association between LUC7L2 expression and patient prognosis, univariate Cox proportional hazards regression and log-rank tests were performed for four survival endpoints: disease-free interval, disease-specific survival, overall survival (OS), and progression-free interval. For PRAD, recurrence-free survival (RFS) was assessed in the TCGA cohort using progression-free interval (PFI) as the recurrence endpoint, whereas relapse-free survival (RFS) endpoints from independent external cohorts were evaluated separately by meta-analysis. Hazard ratios and their 95% confidence intervals were computed for each tumor type to quantify the strength of association between gene expression level and event risk. A hazard ratio > 1 indicated that high LUC7L2 expression was associated with increased event risk, while a hazard ratio < 1 indicated decreased risk. To validate the prognostic association of LUC7L2 in prostate cancer across independent cohorts, a meta-analysis was performed. Publicly available PRAD cohorts with RFS, disease-free survival (DFS), or OS outcomes and corresponding LUC7L2 expression data were included, comprising GSE21034, GSE70768, GSE70769, GSE54460, GSE107299, DKFZ2018, GSE116918, the ICGC-PRAD-CA cohort, and the TCGA-PRAD cohort. For each cohort, the log hazard ratio (logHR) and its standard error were extracted from univariate Cox regression of LUC7L2 expression (high versus low, determined by the optimal cutoff). Between-study heterogeneity was assessed using the Cochran Q test and the I^2^ statistic. Because of substantial between-study heterogeneity, a random-effects (DerSimonian–Laird) model was used to pool effect sizes, and results were presented as pooled HR with 95% confidence intervals.

### 2.4. Pathway Enrichment Analysis

Tumor samples were divided into high-expression (top 30%) and low-expression (bottom 30%) groups based on LUC7L2 expression levels. Differentially expressed genes between the two groups were identified using the limma package (v3.58.1) in R, with the Benjamini–Hochberg method applied to control the false discovery rate. Gene set enrichment *p* values were adjusted using the Benjamini–Hochberg procedure, and gene sets with adjusted *p* < 0.05 were considered significantly enriched. Gene set enrichment analysis was then performed with the clusterProfiler package (v4.10.1) using the Hallmark gene set and the KEGG metabolic gene set. In addition, the GSVA package (v1.50.5) was used to compute Z-scores for 14 functional gene set signatures, including angiogenesis, apoptosis, cell cycle, differentiation, DNA damage, DNA repair, epithelial–mesenchymal transition, hypoxia, inflammation, invasion, metastasis, proliferation, quiescence, and stemness. Pearson correlation analysis was performed to quantify the association between the Z-scores of each gene set and LUC7L2 expression.

### 2.5. Single-Cell and Spatial Transcriptomic Analysis

Expression levels and heterogeneity of LUC7L2 across single-cell subpopulations in PRAD were assessed using the TISCH2 database, analyzing two single-cell transcriptomic datasets (GSE137829 and GSE176031). Spatial transcriptomic data were processed jointly by the Sparkle analysis platform and the SpatialTME tool. Three prostate cancer spatial sections (PRDA1, PRDA2, and PRAD9) were analyzed, with default quality-control filtering (mitochondrial gene threshold and minimum feature cutoff), log-normalization, and the top 2000 highly variable genes used for dimensionality reduction prior to deconvolution and visualization; differential expression between malignant and non-malignant spots was assessed by the Wilcoxon rank-sum test with a significance threshold of adjusted *p* < 0.05. Core cell compositions within each spatial niche were deconvolved using the Spotlight algorithm, and results were visualized with Seurat’s (v5.5.1) SpatialDimPlot function; spatial distribution of gene expression was displayed using SpatialFeaturePlot. To test associations between spatial patterns, the Mantel test was applied. Finally, spatial networks were visualized using LINCET to systematically elucidate the interaction between LUC7L2 expression and the spatial distribution of immune cells.

### 2.6. Cell Culture

The prostate cancer cell lines 22RV1 and LNCaP (ATCC: CRL-2505 and CRL-1740, respectively) were cultured in 1640 basal medium (Servicebio, Wuhan, China, Cat# G4512-500 mL) supplemented with 10% fetal bovine serum (Sangon Biotech, Shanghai, China, Cat# E600001-0500) and 1% penicillin–streptomycin (Servicebio, Cat# G4003-100 mL). All cells were maintained in a humidified incubator at 37 °C with 5% CO_2_.

### 2.7. Quantitative Real-Time PCR

Total RNA was extracted from cells using TRIzol reagent (Cowin Biotech, Shanghai, China, Cat# CW3166M). One microgram of total RNA was reverse-transcribed into complementary DNA using PrimeScript RT Master Mix (novoprotein, Suzhou, China, Cat# E047-01B). The transcript levels of LUC7L2 were then assessed by quantitative real-time PCR using SYBR Green on a CFX Connect™ instrument (Bio-Rad Laboratories, Hercules, CA, USA). Reactions were prepared according to the manufacturer’s instructions for the HiScript II Q RT SuperMix for qPCR kit (Vazyme, Nanjing, China). The amplification program was as follows: initial denaturation at 95 °C for 10 min, followed by 40 cycles of 95 °C for 10 s, 60 °C for 30 s, and 72 °C for 30 s. Primer sequences were as follows: GAPDH-F: GTCTCCTCTGACTTCAACAGCG, GAPDH-R: ACCACCCTGT TGCTGTAGCCAA, LUC7L2-F: GGAGCAGAGAACGATCCAAGAG, LUC7L2-R: TCCGATCTCTGTCACGAGGTGA.

### 2.8. Western Blotting

Western blot analysis was performed on cells grown to confluence in six-well plates. Cells were washed three times with ice-cold phosphate-buffered saline, and total protein was extracted by adding 300 μL of lysis buffer containing 1% (*v*/*v*) protease inhibitor cocktail per well on ice. Protein samples were separated by SDS-polyacrylamide gel electrophoresis and transferred onto polyvinylidene difluoride membranes using a wet transfer system. After transfer, membranes were blocked in protein-free rapid blocking buffer for 30 min at room temperature. Membranes were then incubated overnight at 4 °C with primary antibodies against LUC7L2 (24202-1-AP, Proteintech, Wuhan, China) and β-actin (66009-1-Ig, Proteintech). The following day, after extensive washing, membranes were incubated with appropriate horseradish peroxidase-conjugated secondary antibodies for 1 h at room temperature. Finally, protein bands were visualized using the BeyoECL Plus chemiluminescent substrate (Beyotime, Shanghai, China) and captured with a gel imaging system.

### 2.9. Cell Seeding and Small Interfering RNA Transfection

Cells in the logarithmic growth phase were trypsinized, counted, and resuspended to prepare an appropriate cell suspension. The cells were diluted according to experimental requirements and seeded uniformly into six-well plates at a density of 2 × 10^5^ to 1 × 10^6^ cells per well; plates were labeled accordingly. When cells reached approximately 70% confluence, transfection was performed. First, the original culture medium was discarded and replaced with 1 mL of serum-free basal medium per well. Two sterile EP tubes were prepared, each containing 125 μL of Opti-MEM (Gibco, Grand Island, NY, USA) reduced-serum medium. To one tube, 10 μL of Melun high-efficiency transfection reagent was added, and to the other, 2.5 μg of the target small interfering RNA (three independent siRNAs targeting LUC7L2 (si-LUC7L2-1, si-LUC7L2-2, and si-LUC7L2-3) plus a non-targeting negative control siRNA (si-NC)). Both tubes were gently mixed and incubated at room temperature for 5 min. After incubation, the contents of the two tubes were combined, gently mixed again, and incubated at room temperature for an additional 15 min to allow stable transfection complex formation. Transfected cells were immediately returned to the incubator. At 4–6 h post-transfection, 1 mL of complete culture medium containing 20% fetal bovine serum was added to each well. Cells were collected for subsequent experimental analysis 48 h after transfection. The most efficient siRNA was selected for subsequent functional assays, and all three independent siRNAs yielded consistent results. The siRNA sequences are provided in Supplementary Table S1.

### 2.10. Cell Counting Kit-8 Assay, EdU Proliferation Assay, and Cell Cycle Analysis

Cells in the logarithmic growth phase were counted and seeded into 96-well plates at a density of 6000 cells per well; plates were labeled accordingly. Seeded plates were placed in a humidified incubator at 37 °C with 5% CO_2_ and allowed to attach. Subsequently, 10 μL of Cell Counting Kit-8 reagent was added to each well. Plates were gently shaken to mix and then incubated in the dark for 2 h. After incubation, absorbance at 450 nm was measured using a microplate reader. The obtained values reflect cellular metabolic activity and indirectly indicate cell viability in each group. All procedures were performed under light-protected conditions to minimize photodegradation. Cell proliferation was additionally assessed by a 5-ethynyl-2′-deoxyuridine (EdU) incorporation assay. Cells were incubated with 10 μM EdU for 2 h, then fixed, permeabilized, and labeled using a Click-iT EdU detection kit according to the manufacturer’s instructions. The proportion of EdU-positive (proliferating) cells was quantified by flow cytometry. For cell cycle distribution analysis, cells were collected, fixed in ice-cold 70% ethanol overnight at 4 °C, washed with phosphate-buffered saline, and stained with propidium iodide (50 μg/mL) in the presence of RNase A for 30 min at 37 °C in the dark. DNA content was then measured by flow cytometry, and the percentages of cells in the G0/G1, S, and G2/M phases were quantified using flow cytometric analysis software (FCS Express, v7.18.0015).

### 2.11. Flow Cytometry for Apoptosis Detection

Transfected cells were collected and subjected to apoptosis induction where applicable. After centrifugation at 300× *g* for 5 min, the supernatant was discarded, and the cell pellet was gently washed once with ice-cold phosphate-buffered saline. Following another centrifugation, the pellet was resuspended in an appropriate volume of PBS and counted. A total of 1–5 × 10^5^ cells were aliquoted into a centrifuge tube, centrifuged again at 300× *g* for 5 min, and the supernatant was thoroughly removed. The cells were washed once with PBS, then resuspended in 200 μL of 1× Annexin V binding buffer (diluted to working concentration). Sequentially, 5 μL of Annexin V-FITC and 10 μL of propidium iodide were added. The samples were gently vortexed and incubated in the dark at room temperature for 15–20 min to ensure sufficient dye binding. After incubation, 300 μL of 1× Annexin V binding buffer was added to each tube and mixed gently to terminate the staining reaction. Stained samples were immediately analyzed by flow cytometry to evaluate apoptosis and necrosis.

### 2.12. Transwell Invasion/Migration Assay

Forty-eight hours after transfection, cells were trypsinized, counted, and diluted to appropriate densities. For invasion assays, the upper chambers (0.3 mL, serum-free medium) were pre-coated with Matrigel. Cells were seeded at a density of 3 × 10^4^ to 1 × 10^5^ cells per chamber. The lower chambers were filled with 0.5 mL of complete medium containing serum. The plates were incubated at 37 °C in a 5% CO_2_ incubator. After incubation, cells on the lower surface of the membrane were fixed with 4% paraformaldehyde for 15 min at room temperature, stained with 0.1% crystal violet for 5 min, rinsed with PBS, and air-dried. Images were captured under a microscope.

### 2.13. Senescence-Associated β-Galactosidase (SA-β-Gal) Staining

After cells reached confluence, the culture medium was removed, and the cell layer was gently rinsed once with ice-cold PBS. Sufficient SA-β-gal fixation solution (typically containing 2% formaldehyde and 0.2% glutaraldehyde in buffer) was added to each well and incubated at room temperature for 15 min to stabilize cell structure. After fixation, the solution was thoroughly aspirated, and the cells were washed three times with PBS, 3 min each, to remove residual fixative. Freshly prepared SA-β-gal staining working solution was then added to each well. The working solution contained 1 mg/mL X-gal substrate, 5 mM potassium ferricyanide, 5 mM potassium ferrocyanide, 2 mM magnesium chloride, and 40 mM citric acid–phosphate buffer (pH 6.0). To prevent evaporation during prolonged incubation, the plate was sealed with parafilm or plastic wrap and incubated overnight at 37 °C. The next day, the staining solution was carefully removed, and the cell layer was washed twice with PBS and once with distilled water to remove residual salts and unbound substrate. After air-drying, the plate was examined under an inverted microscope. Senescent cells, which exhibit SA-β-gal activity, hydrolyze X-gal to produce a blue product, appearing as characteristic blue staining. The staining pattern was used to assess cellular senescence and compare differences between experimental groups.

### 2.14. Immunofluorescence Staining

Cells were fixed with 4% paraformaldehyde for 15 min, washed with PBS, and blocked with 3% bovine serum albumin at 37 °C for 30 min. Subsequently, cells were incubated with anti-γ-H2AX primary antibody (1:200 dilution) overnight at 4 °C. After washing with PBS, cells were incubated with fluorescently conjugated secondary antibody (1:200, 37 °C, 30 min) in the dark. Nuclei were counterstained with DAPI for 3 min. Coverslips were mounted, and images were acquired using a fluorescence microscope. DAPI-stained nuclei appeared blue, and γ-H2AX-positive foci appeared red, reflecting the extent of DNA damage.

### 2.15. Statistical Analysis

All statistical analyses were performed using GraphPad Prism 10.4.2 software, while data visualization and bioinformatics analyses were conducted in R (version 4.3.2). To ensure reliability and reproducibility, each experiment was independently repeated at least three times (i.e., three or more biological replicates), and final data are presented as arithmetic mean ± standard deviation. Differences between groups were evaluated using the two-tailed unpaired Student’s *t*-test; a *p* value less than 0.05 was considered statistically significant. For high-dimensional correlation screens (e.g., the pan-cancer genomic-instability correlations and the 1503-transcription-factor analysis), the Benjamini–Hochberg FDR method was applied, and FDR < 0.05 was considered significant. For data that did not meet the parametric assumptions, the Mann–Whitney U nonparametric test was applied as appropriate. All figures were prepared according to journal submission guidelines, and error bars uniformly represent standard deviation to ensure conservative statistical inference.

## 3. Results

### 3.1. Pan-Cancer Expression Profiling of LUC7L2

**Figure 1:**
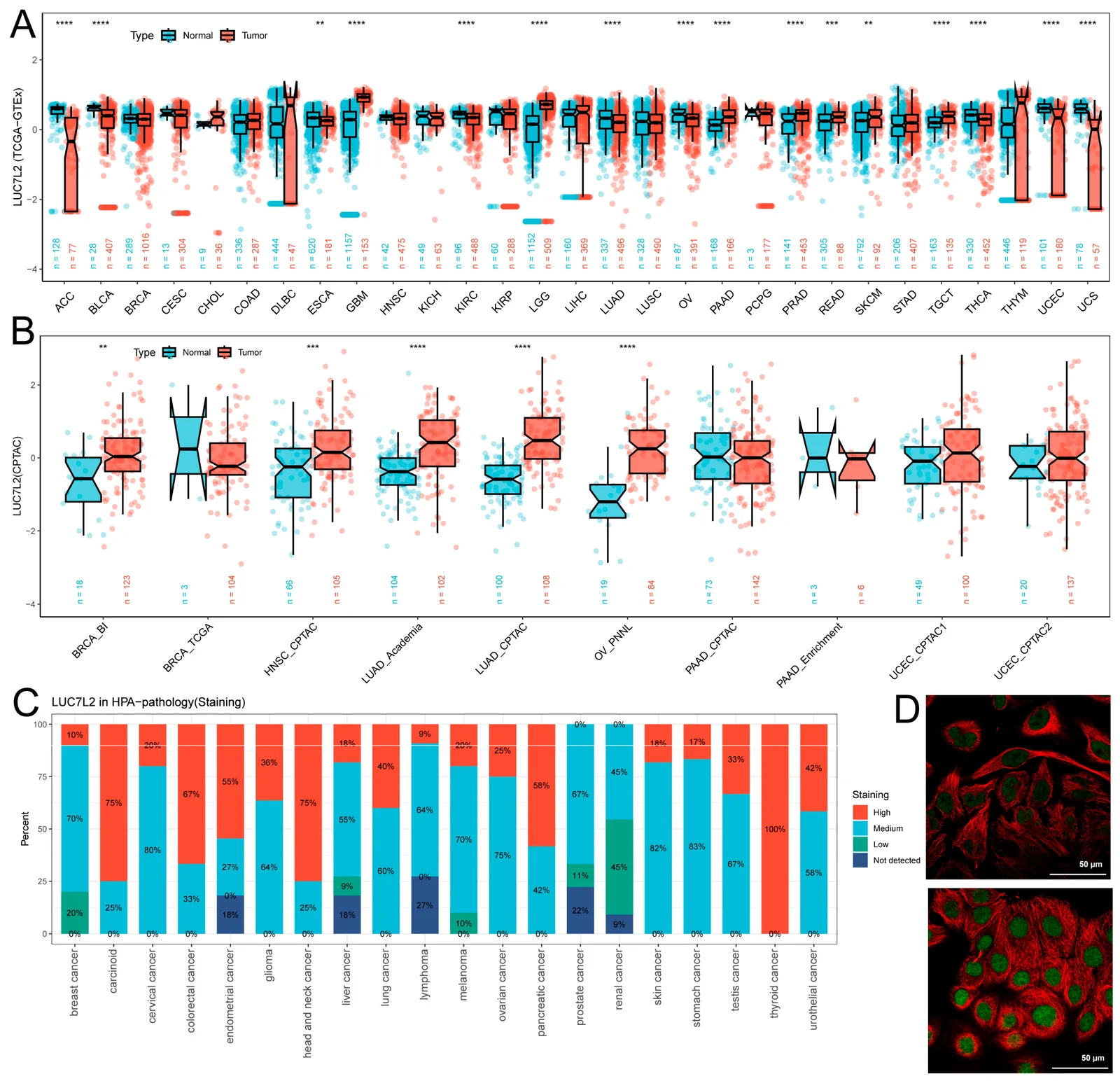
Pan-cancer expression profiling of LUC7L2. (**A**) mRNA expression levels of LUC7L2 across multiple solid tumor types and corresponding normal tissues in the TCGA and GTEx databases. (**B**) Protein expression levels of LUC7L2 in paired tumor and normal tissues from the CPTAC database. (**C**) Immunohistochemistry staining intensity of LUC7L2 across various cancer types in the Human Protein Atlas (HPA) database. (**D**) Immunofluorescence staining of LUC7L2 (green) in HeLa and MCF-7 cells showed predominant nuclear localization, with microtubules shown in red, showing predominant nuclear localization. ** *p* < 0.01, *** *p* < 0.001, **** *p* < 0.0001.

To systematically characterize LUC7L2 expression across multiple malignancies, we performed differential expression analysis at the transcriptional level using RNA-Seq data from the TCGA and GTEx databases. Compared with normal tissues, LUC7L2 was significantly upregulated in glioblastoma multiforme (GBM), lower-grade glioma (LGG), prostate adenocarcinoma (PRAD), rectal adenocarcinoma (READ), skin cutaneous melanoma (SKCM), and testicular germ cell tumor (TGCT). Conversely, its expression was markedly downregulated in adrenocortical carcinoma (ACC), bladder urothelial carcinoma (BLCA), esophageal carcinoma (ESCA), kidney renal clear cell carcinoma (KIRC), lung adenocarcinoma (LUAD), ovarian serous cystadenocarcinoma (OV), thyroid papillary carcinoma (THCA), uterine corpus endometrial carcinoma (UCEC), and uterine carcinosarcoma (UCS) (Figure 1A). At the protein level, analysis of CPTAC proteomic data further confirmed the upregulation of LUC7L2 in breast cancer (BRCA), head and neck squamous cell carcinoma (HNSC), lung adenocarcinoma (LUAD), and ovarian cancer (OV) (Figure 1B).

Immunohistochemistry scores from The Human Protein Atlas (HPA) database revealed relatively high staining intensity of LUC7L2 in carcinoid tumors, colorectal cancer, endometrial cancer, head and neck cancer, pancreatic cancer, and thyroid cancer (Figure 1C). Finally, immunofluorescence staining in the HeLa and MCF-7 cell lines showed that the LUC7L2 protein predominantly localizes to the nucleus, suggesting its involvement in nuclear regulatory processes (Figure 1D).

### 3.2. Diagnostic Value and Mutation Analysis of LUC7L2

**Figure 2:**
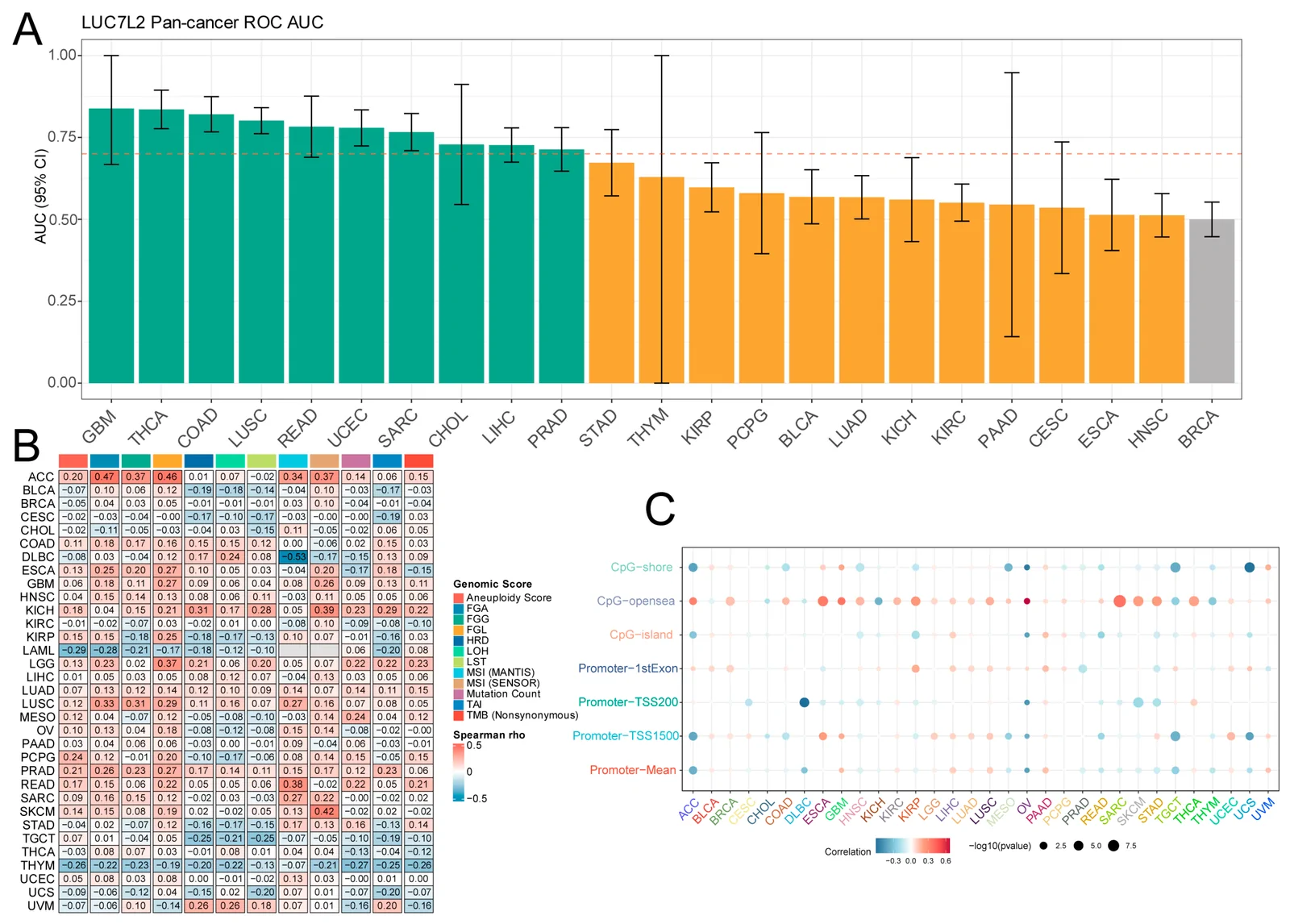
Diagnostic value, mutation, and methylation analysis of LUC7L2 in pan-cancer. (**A**) Receiver operating characteristic (ROC) curve analysis evaluating the ability of LUC7L2 expression to distinguish tumor from normal tissues across multiple cancer types. (**B**) Correlation of LUC7L2 expression with gene mutation status, microsatellite instability (MSI), and tumor mutational burden (TMB) in pan-cancer. (**C**) Association between LUC7L2 expression and DNA methylation levels across various cancer types.

To comprehensively evaluate the diagnostic potential of LUC7L2, receiver operating characteristic (ROC) curves were generated. LUC7L2 expression exhibited excellent diagnostic performance in glioblastoma multiforme (GBM), thyroid papillary carcinoma (THCA), colon adenocarcinoma (COAD), lung squamous cell carcinoma (LUSC), rectal adenocarcinoma (READ), endometrial carcinoma (UCEC), soft tissue sarcoma (SARC), cholangiocarcinoma (CHOL), hepatocellular carcinoma (LIHC), and prostate adenocarcinoma (PRAD), with area under the curve (AUC) values exceeding 0.7 in all cases (Figure 2A). Genomic instability scores are critical indicators of tumor genomic disarray and guide precision therapeutic strategies. We therefore examined the association between LUC7L2 expression and genomic instability. LUC7L2 showed strong correlations with microsatellite instability (MSI) and tumor mutational burden (TMB) across multiple cancer types (Figure 2B), implicating its role in the maintenance of genomic stability.

Furthermore, the relationship between DNA methylation—an essential epigenetic mechanism—and LUC7L2 expression was investigated. While LUC7L2 expression was positively correlated with methylation levels at CpG open-sea regions in several tumors, it was significantly negatively correlated with methylation at promoter TSS200 regions (Figure 2C). These findings suggest that LUC7L2 expression may be negatively regulated by promoter methylation, whereas open-sea methylation may modulate its expression through indirect mechanisms.

### 3.3. Prognostic Significance of LUC7L2

**Figure 3:**
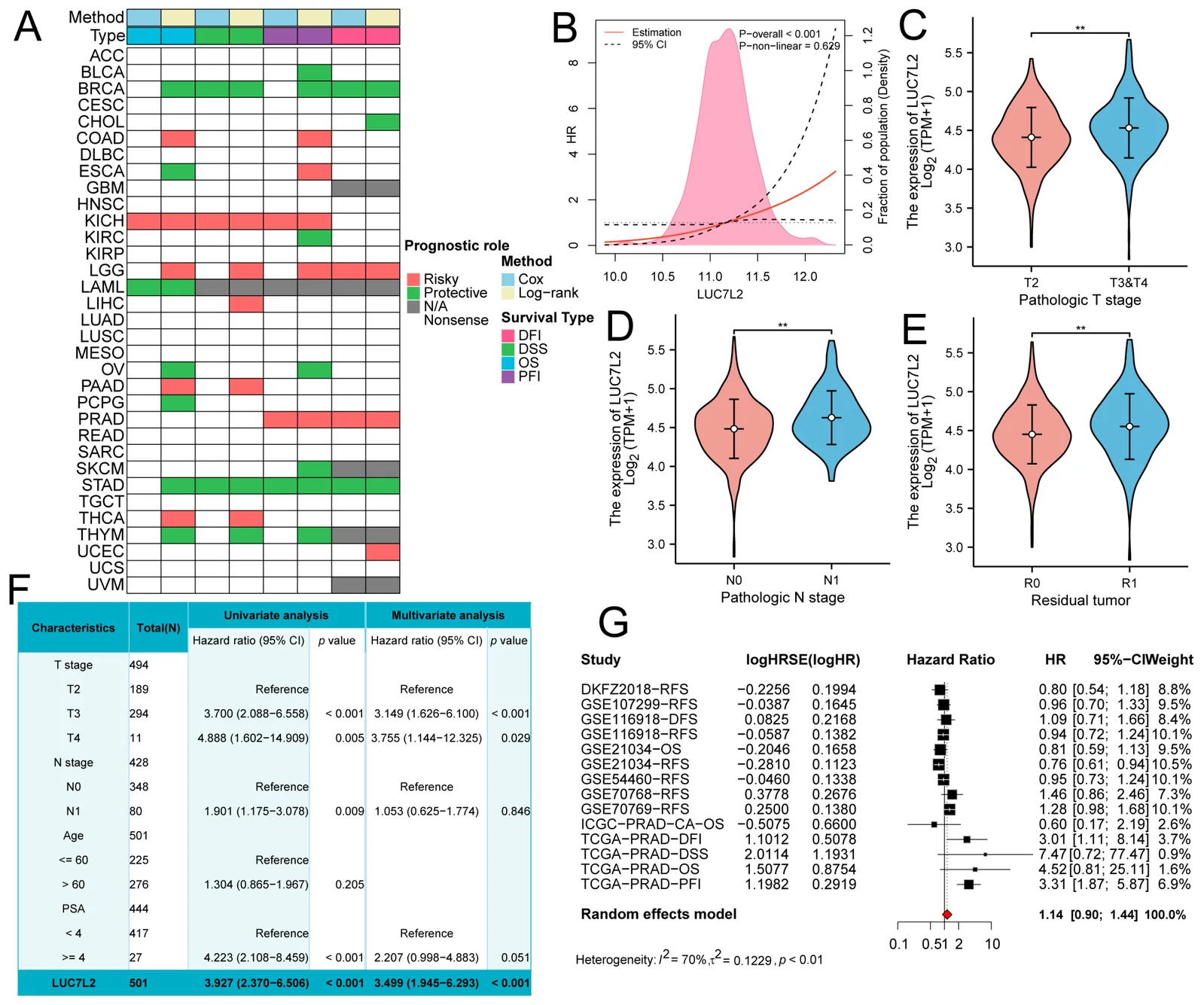
Prognostic analysis of LUC7L2 in pan-cancer. (**A**) Forest plot summarizing the hazard ratios (HRs) of LUC7L2 for overall survival or progression-free survival across multiple cancer types based on Cox proportional hazards regression and log-rank tests. (**B**) Restricted cubic spline Cox regression model illustrating the relationship between LUC7L2 expression (x-axis) and hazard ratio (y-axis) in prostate adenocarcinoma (PRAD). Solid line represents the estimated HR; shaded area indicates 95% confidence interval; horizontal dashed line marks HR = 1. The rug plot at the bottom shows the distribution of LUC7L2 expression values, and the background density plot reflects the kernel density estimation. (**C**–**E**) Box plots showing the LUC7L2 expression levels in PRAD according to T stage (**C**), N stage (**D**), and surgical margin status (**E**). (**F**) Univariate and multivariate Cox regression analyses of LUC7L2 and other clinical covariates for recurrence-free survival in PRAD. (**G**) Meta-analysis of the prognostic value of LUC7L2 in multiple independent PRAD cohorts, with pooled HR and 95% CI. ** *p* < 0.01.

To systematically evaluate the association between aberrant LUC7L2 expression and patient outcomes, we performed Cox proportional hazards regression and log-rank tests using TCGA cohort data. LUC7L2 emerged as a protective factor in breast cancer (BRCA), acute myeloid leukemia (LAML), ovarian serous cystadenocarcinoma (OV), stomach adenocarcinoma (STAD), and thymoma (THYM), but as a risk factor in kidney chromophobe carcinoma (KICH), lower-grade glioma (LGG), prostate adenocarcinoma (PRAD), pancreatic adenocarcinoma (PAAD), thyroid papillary carcinoma (THCA), and hepatocellular carcinoma (LIHC). Given the inverse relationship between LUC7L2 expression and prognosis in PRAD, we focused subsequent analyses on this cancer type (Figure 3A).

A restricted cubic spline (RCS) model revealed a linear dose–response relationship between LUC7L2 expression and progression-free interval (PFI), indicating that higher expression levels correspond to a linearly increasing risk of recurrence in PRAD patients (Figure 3B). LUC7L2 mRNA transcript abundance progressively increased with advancing pathologic T and N stage (Figure 3C,D) and was significantly higher in patients with residual tumor (R1) than in those with negative surgical margins (R0) (Figure 3E). Both univariate and multivariate Cox regression analyses confirmed that LUC7L2 remained an independent predictor of progression-free interval in PRAD after adjusting for pathologic T stage, N stage, age, and preoperative PSA level (Figure 3F). To validate the external reproducibility of these findings, a random-effects meta-analysis integrating multiple independent PRAD cohorts yielded a pooled hazard ratio of 1.14 (95% CI: 0.90–1.44), with substantial between-study heterogeneity (I^2^ = 70%, *p* < 0.01), indicating a consistent trend toward worse outcome that was driven primarily by the TCGA-PRAD progression-free interval (Figure 3G).

### 3.4. Single-Cell Analysis of LUC7L2 Expression in Prostate Cancer

**Figure 4:**
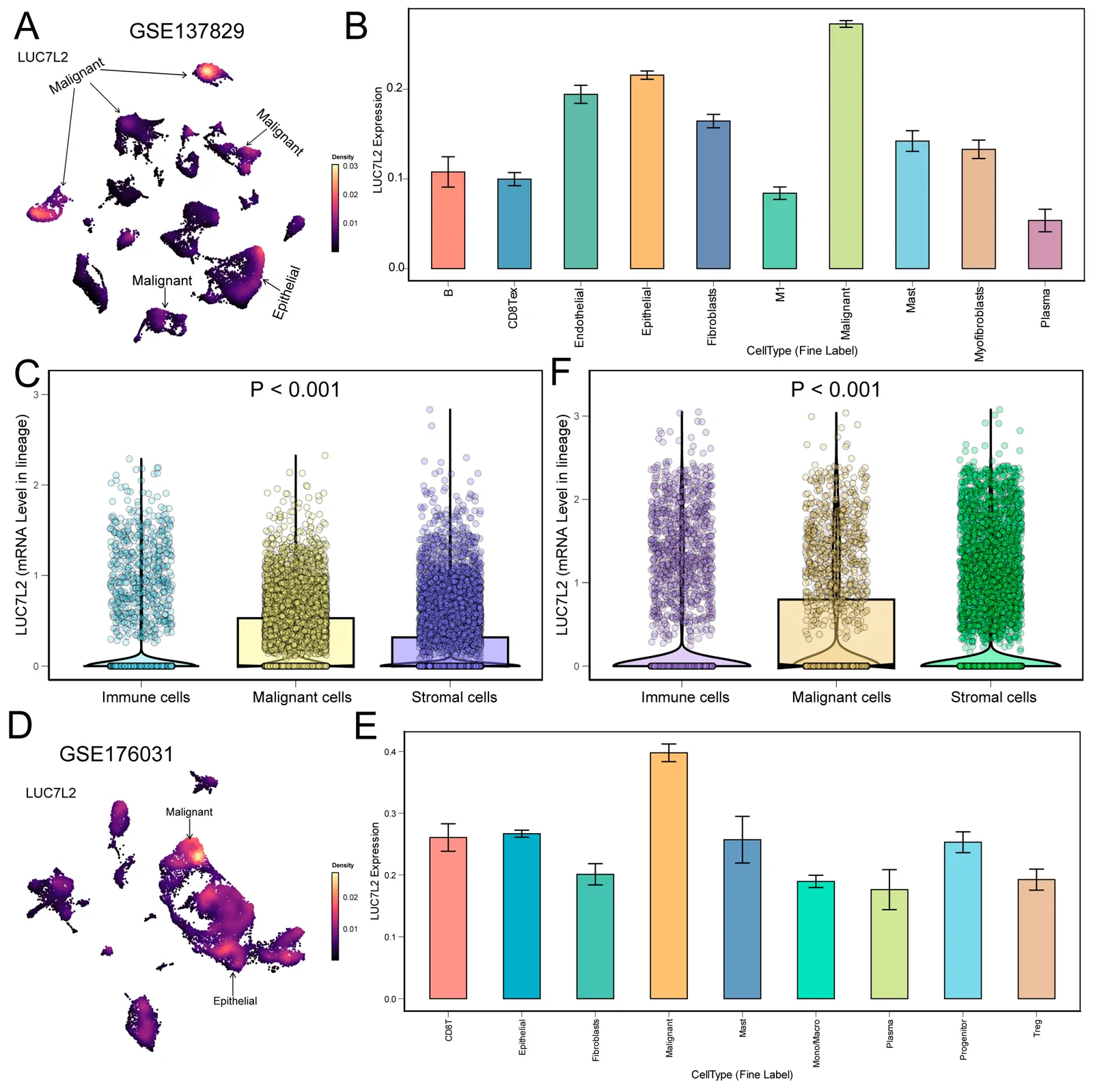
Single-cell analysis of LUC7L2 expression in prostate adenocarcinoma (PRAD). (**A**) UMAP density plots showing LUC7L2 expression in malignant and epithelial cells in the GSE137829 cohort. (**B**) Expression levels of LUC7L2 across different cell types in the GSE137829 cohort. (**C**) Differential expression analysis of LUC7L2 among malignant, immune, and stromal cells in the GSE137829 cohort. (**D**) UMAP density plots showing LUC7L2 expression in malignant and epithelial cells in the GSE176031 cohort. (**E**) Expression levels of LUC7L2 across different cell types in the GSE176031 cohort. (**F**) Differential expression analysis of LUC7L2 among malignant, immune, and stromal cells in the GSE176031 cohort.

To characterize LUC7L2 expression at a single-cell resolution, we analyzed two independent scRNA-Seq datasets from TISCH2 (PRAD_GSE137829 and PRAD_GSE176031). In the GSE137829 cohort, UMAP projection and averaged expression profiles across cell types revealed that LUC7L2 was preferentially expressed in malignant cells (Figure 4A,B). Its transcript levels were significantly elevated in malignant cells compared with immune and stromal populations (Figure 4A–C). A similar expression pattern was observed in the GSE176031 dataset (Figure 4D–F). These findings collectively suggest that LUC7L2 may play a functional role specifically within malignant cells of prostate cancer.

### 3.5. Spatial Transcriptomic Profiling

**Figure 5:**
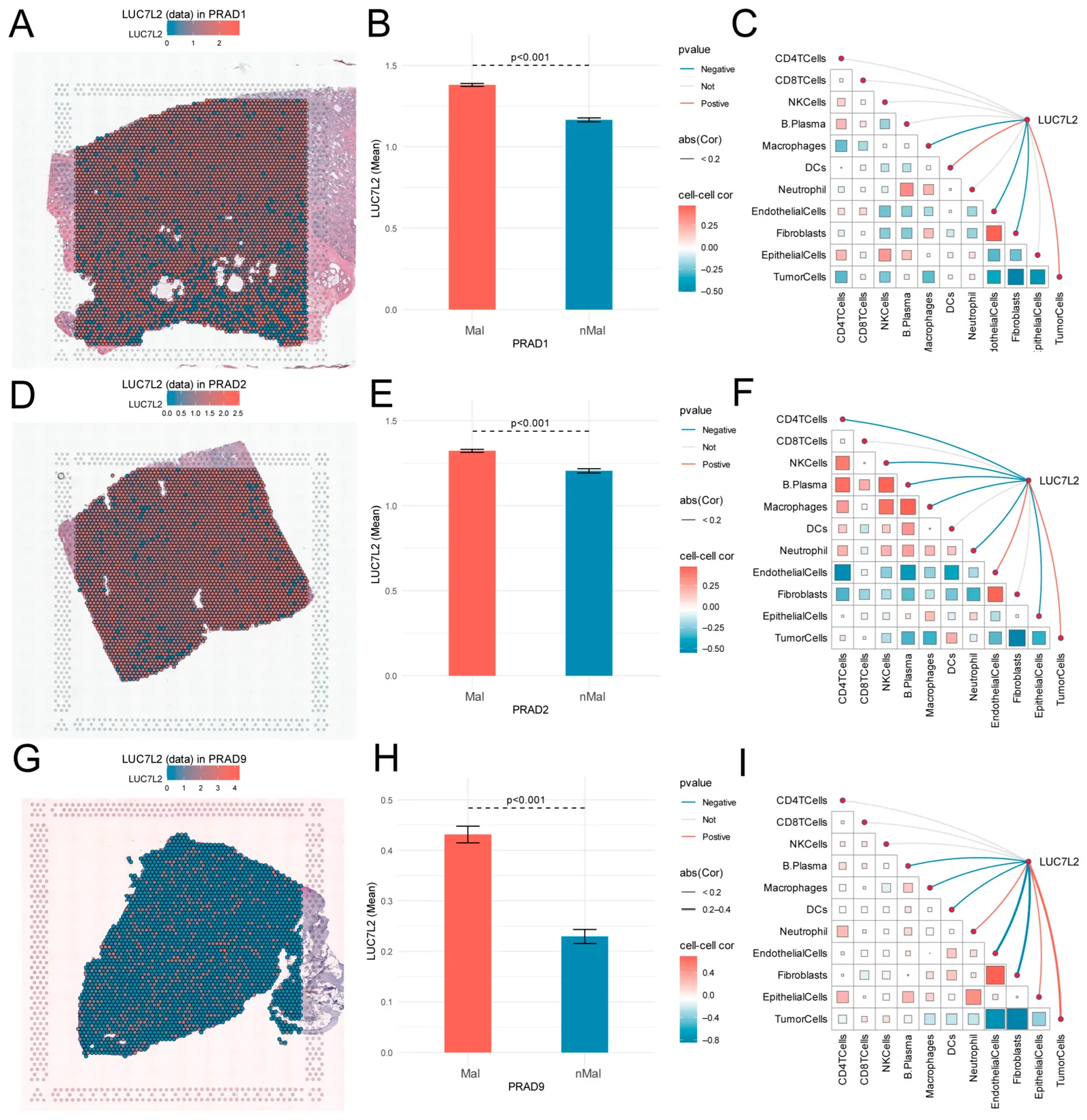
Spatial transcriptomic analysis of LUC7L2 expression in prostate cancer. (**A**–**C**) Spatial profiling of LUC7L2 in the PRDA1 section. (**A**) SpatialFeaturePlot showing the distribution of LUC7L2 transcripts across the tissue section. (**B**) Comparison of LUC7L2 expression between malignant and non-malignant regions in the PRDA1 section. (**C**) Spatial correlation analysis between LUC7L2 expression and cell-type proportions across all capture spots in the PRDA1 section. (**D**–**F**) Corresponding analyses for the PRDA2 tissue section. (**D**) Spatial expression pattern of LUC7L2. (**E**) Differential expression between malignant and non-malignant regions. (**F**) Correlation of LUC7L2 expression with cell-type composition. (**G**–**I**) Corresponding analyses for the PRAD9 tissue section. (**G**) Spatial localization of the LUC7L2 transcripts. (**H**) Expression difference between malignant and non-malignant areas. (**I**) Spatial association between LUC7L2 expression and cell-type proportions.

Spatial transcriptomic analysis using the SpatialFeaturePlot visualization showed that the spatial distribution of LUC7L2 transcripts closely aligned with the localization of tumor cells. High-signal regions predominantly corresponded to tumor-dense microenvironments, whereas expression in normal tissue regions was markedly lower. Moreover, the signal intensity of LUC7L2 increased with the proportion of tumor cells in individual capture spots (Figure 5A,B,D,E,G,H). Consistently, LUC7L2 expression was significantly higher in malignant than in non-malignant regions (Figure 5C,F,I), suggesting that its upregulation may be specific to tumor areas and contribute to the maintenance and regulation of the malignant microenvironment.

### 3.6. Pathway Correlation and Interactome Analysis

**Figure 6:**
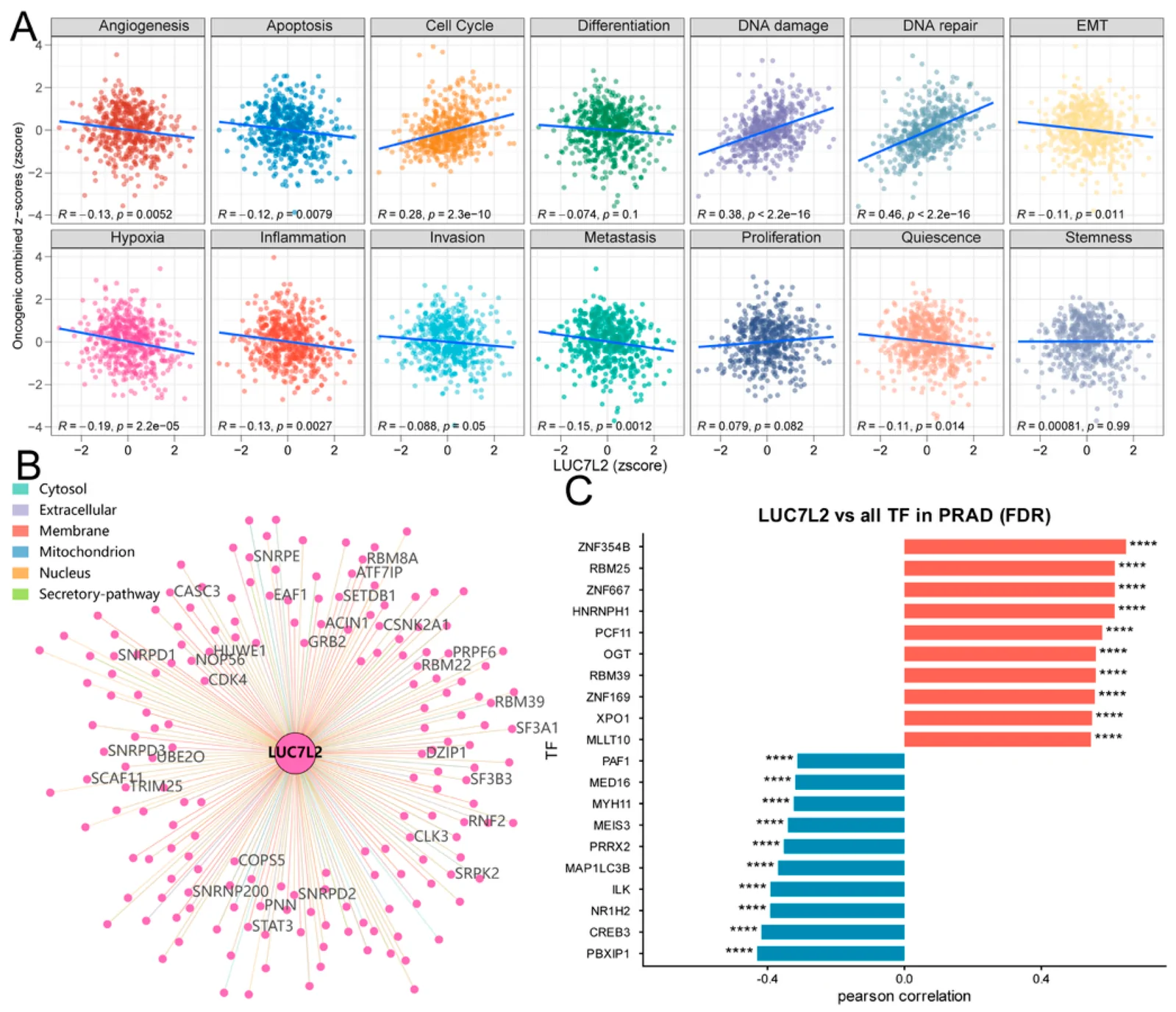
Pathway and potential interacting molecule analysis of LUC7L2 in prostate cancer. (**A**) Scatter plot showing the correlation between LUC7L2 expression (z-score, x-axis) and a combined z-score of tumor-associated features (y-axis). A linear regression line is fitted, with Pearson correlation coefficient and *p*-value displayed. (**B**) Subcellular localization interaction network based on the comPPI database. The central node represents LUC7L2, and surrounding nodes represent candidate proteins with reported interactions. (**C**) Pearson correlation analysis between LUC7L2 expression and each transcription factor (TF), with correlation coefficients calculated individually. **** *p* < 0.0001.

To delineate the functional landscape of LUC7L2 across cancers, we employed GSVA combined with Z-score normalization to evaluate its association with 14 tumor-related pathways. LUC7L2 expression showed robust correlations with cell cycle and DNA damage repair processes (Figure 6A). Screening for potential interacting molecules identified NOP56, LUC7L3, ITPRID2, and ANTKMT as candidate partners (Figure 6B). In the PRAD samples, we systematically assessed the correlation between LUC7L2 expression and the expression of 1503 transcription factors (TFs) using Pearson correlation analysis (Figure 6C). Among these, 798 TFs were significantly positively correlated with LUC7L2 (*p* < 0.05), and 266 were significantly negatively correlated (*p* < 0.05), with ZNF354B showing the strongest positive correlation.

### 3.7. Functional Validation of LUC7L2 in Prostate Cancer Cells

**Figure 7:**
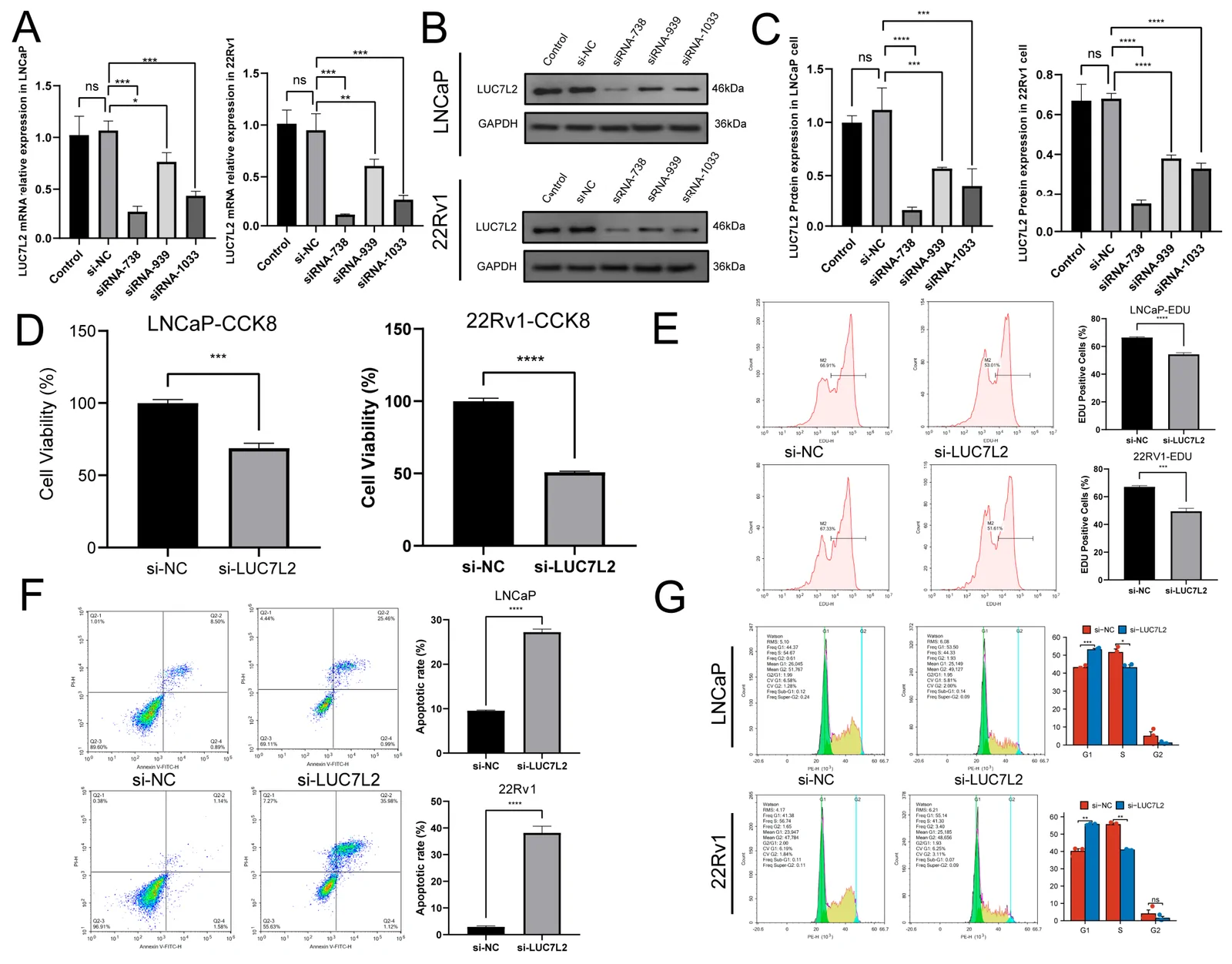
Effect of LUC7L2 knockdown on prostate cancer cell functions. (**A**) RT-PCR validation of LUC7L2 knockdown efficiency. (**B**) Western blot analysis of LUC7L2 protein levels after knockdown. (**C**) Quantification of Western blot results for LUC7L2 knockdown efficiency. (**D**) CCK-8 assay assessing cell proliferation following LUC7L2 knockdown. (**E**) EdU flow cytometry assay evaluating cell proliferation after LUC7L2 knockdown. (**F**) Flow cytometry analysis of apoptosis after LUC7L2 knockdown. (**G**) Flow cytometry analysis of cell cycle distribution after LUC7L2 knockdown. ns, not significant, * *p* < 0.05, ** *p* < 0.01, *** *p* < 0.001, **** *p* < 0.0001.

**Figure 8:**
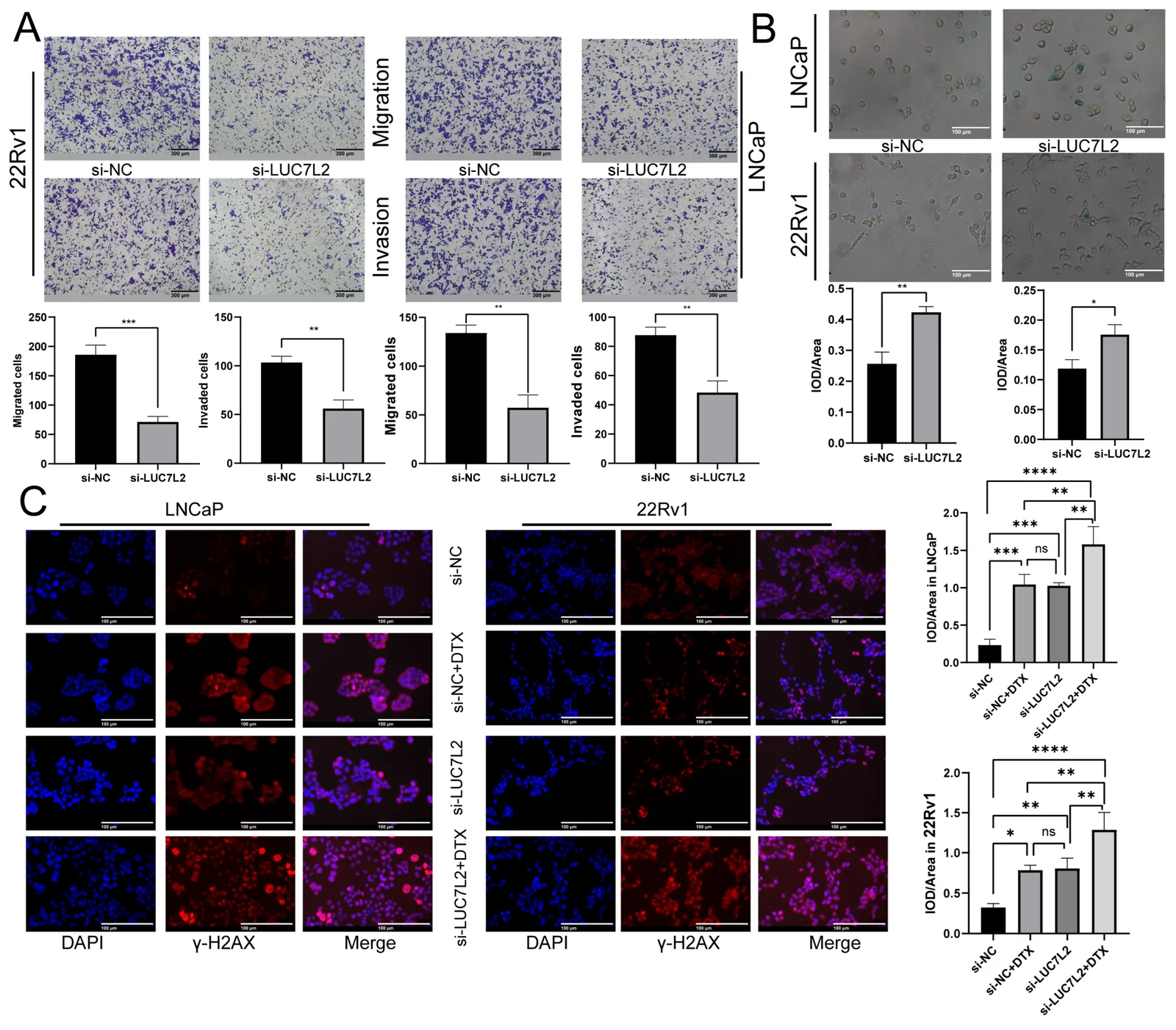
Effects of LUC7L2 knockdown on the invasion and DNA damage of prostate cancer cells. (**A**) Effect of LUC7L2 knockdown on cell migration and invasion in LNCaP and 22RV1 cells. (**B**) β-Galactosidase staining assessing cellular senescence in LNCaP and 22RV1 cells after LUC7L2 knockdown. (**C**) Immunofluorescence staining of γ-H2AX (a marker of DNA damage) in LNCaP and 22RV1 cells after LUC7L2 knockdown. Scale Bar: 100 µm. * *p* < 0.05, ** *p* < 0.01, *** *p* < 0.001, **** *p* < 0.0001.

To investigate the functional role of LUC7L2 in prostate cancer, we designed specific siRNAs targeting its transcript and validated knockdown efficiency in LNCaP and 22Rv1 cells using RT-PCR and Western blot (Figure 7A–C). The most efficient siRNA was selected for subsequent experiments. CCK-8 and EdU assays both demonstrated that LUC7L2 knockdown significantly suppressed cell proliferation (Figure 7D,E). Flow cytometry revealed a marked increase in apoptosis and S-phase cell cycle arrest upon knockdown (Figure 7F,G). Transwell assays further indicated that both migration and invasion capacities were substantially impaired (Figure 8A). Additionally, β-galactosidase staining showed enhanced cellular senescence, and γ-H2AX immunofluorescence indicated increased DNA damage (Figure 8B,C). Collectively, these results establish LUC7L2 as a critical regulator of prostate cancer cell proliferation, apoptosis, cell cycle progression, migration, invasion, senescence, and DNA damage repair.

## 4. Discussion

LUC7L2 frequently undergoes expression dysregulation and genetic alterations across various cancers, with functional roles that exhibit marked tissue specificity. By integrating multi-platform data, this study systematically characterizes the tissue-specific expression landscape of LUC7L2 across multiple cancer types: it is significantly upregulated in GBM and PRAD, but downregulated in KIRC and LUAD. This pattern suggests that LUC7L2 may exert dual or even opposing regulatory functions in different tumor microenvironments.

ROC analysis showed that LUC7L2 achieved an AUC exceeding 0.7 in 10 cancer types, including GBM, THCA, and COAD, indicating its potential as a pan-cancer diagnostic marker. This finding aligns with previous studies: in high-grade serous ovarian cancer, LUC7L2 has been incorporated into a 10-gene diagnostic signature that effectively distinguishes malignant from benign gynecologic diseases [20]. Moreover, large-scale CRISPR screens have identified it as a selective dependency gene in chordoma [21], further supporting its potential as both a diagnostic and therapeutic target. Nonetheless, the diagnostic performance requires validation in large, independent external cohorts to assess its robustness. Regarding prognosis, this study confirms that LUC7L2 is an independent prognostic risk factor in prostate cancer. Its expression increases with TNM stage progression and biochemical recurrence, and this finding was validated through multi-cohort meta-analysis. LUC7L2 has been identified as a splicing factor significantly correlated with overall survival and included in a prognostic splicing regulatory network [22]. Together with our findings in PRAD, LUC7L2 has the potential to serve as a novel molecular indicator for prognostic stratification across multiple cancers, particularly for guiding the precision management of prostate cancer patients.

Promoter TSS200 methylation is significantly negatively correlated with LUC7L2 expression, while CpG open sea methylation shows a positive correlation, revealing a dual mode of epigenetic regulation on this gene’s transcription. Multiple genetic association studies have reported that polymorphisms in the LUC7L2 region are associated with uric acid response to hydrochlorothiazide treatment in hypertensive patients and with potential hypertension susceptibility in the Jordanian population [23,24]. Multi-omics Mendelian randomization has identified it as a lactylation-related gene negatively associated with the risk of intervertebral disc degeneration [25]. Genomic analysis of pediatric pilocytic astrocytoma has placed LUC7L2 within a key gene network in the BRAF fusion region, potentially influencing tumor growth together with MAPK signaling and retinoic acid-mediated apoptosis [26]. These lines of evidence provide new molecular insights into the epigenetic basis of LUC7L2’s tissue-specific expression.

In vitro functional experiments confirm that LUC7L2 knockdown significantly inhibits the proliferation, migration, and invasion of prostate cancer cells and induces apoptosis, senescence, and DNA damage. These results are highly consistent with pathway enrichment analyses showing the activation of cell cycle and DNA damage repair pathways, forming a complete chain of evidence from bioinformatics to functional validation. Similarly, in ovarian cancer, LUC7L2, as a member of the splicing factor family, has been found to be significantly associated with patient survival [27]. Furthermore, we observed potential interactions between LUC7L2 and the splicing factor LUC7L3 as well as the nucleolar protein NOP56. Combined with its nuclear localization, this suggests that LUC7L2 may influence tumor phenotypes through nuclear processes such as RNA splicing or ribosome biogenesis. The specific molecular mechanisms remain to be elucidated through experiments such as co-immunoprecipitation and RNA-Seq.

This study has several limitations: functional validation was limited to prostate cancer cell lines and has not been performed in in vivo animal models or other cancer types; single-cell analyses relied on public databases without independent cohort validation; and the knockdown experiments lacked rescue controls, which cannot fully exclude off-target effects. Future studies should include in vivo tumor formation experiments, functional comparisons across multiple cancer types, and CRISPR knockout validation to comprehensively evaluate the feasibility of LUC7L2 as a therapeutic target.

In summary, this study systematically portrays the pan-cancer expression landscape of LUC7L2 through multi-omics integrative analysis and establishes it as a potential functional regulator and independent poor prognostic marker in prostate cancer. We found that high LUC7L2 expression is closely associated with genomic instability and specific methylation patterns, and it is specifically enriched in malignant cell subpopulations at both single-cell and spatial levels. Functional experiments demonstrate that the targeted inhibition of LUC7L2 effectively blocks prostate cancer cell proliferation, induces senescence and apoptosis, and exacerbates DNA damage, highlighting its potential as a candidate therapeutic target that warrants further in vivo validation. These findings offer new perspectives for precision risk stratification in prostate cancer and lay a theoretical foundation for developing anti-tumor strategies targeting RNA splicing factors.

## Data Availability

The single-cell transcriptomic profiles generated during this work are accessible in the Gene Expression Omnibus (GEO) repository under accession identifiers GSE137829 and GSE176031. Bulk transcriptome data and corresponding clinical metadata were obtained from The Cancer Genome Atlas portal, which can be accessed at https://portal.gdc.cancer.gov/analysis_page?app=Projects (accessed on 9 May 2026).

## Supplementary Materials

The following supporting information can be downloaded at: https://www.mdpi.com/article/10.3390/biomedicines14102294/s1, Table S1. Small interfering RNA (siRNA) sequences used in this study.

## References

[1] Raychaudhuri R., Lin D.W., Montgomery R.B. (2025). Prostate Cancer: A Review. JAMA.

[2] Paralkar D., Akbari A., Aron M. (2024). Prostatic adenocarcinoma: Molecular underpinnings and treatment-related options. Urol. Oncol..

[3] Li S., Liu Y., Wu X., Zhang Q., Yuan W. (2025). UBE2C: A potential therapeutic target and prognostic biomarker for prostate cancer patients. Funct. Integr. Genomics.

[4] Tang R., Wang C., Zhu Y., Liu K., Wu Z. (2025). SLC45A2 drives prostate cancer progression through tumor promotion and immune suppression. Sci. Rep..

[5] Gao H.T., Yang Z., Sun H., Zhang Y., Wang Z., Liu W.Y., Wen H.Z., Qu C.B., Wang X.L. (2023). TREM2 as an independent predictor of poor prognosis promotes the migration via the PI3K/AKT axis in prostate cancer. Am. J. Transl. Res..

[6] Udensi U.K., Tchounwou P.B. (2016). Oxidative stress in prostate hyperplasia and carcinogenesis. J. Exp. Clin. Cancer Res..

[7] Di Carlo E., Sorrentino C. (2024). Oxidative Stress and Age-Related Tumors. Antioxidants.

[8] Krishna M., Garg P., Horne D., Salgia R., Singhal S.S. (2026). Inflammation-driven transcriptional reprogramming in prostate cancer: Convergence of NFkappaB, JAK/STAT3, and epigenetic remodeling in castration-resistant disease. Cancer Lett..

[9] Kurt H.A., Kilicarslan S., Cicekliyurt M.M., Kilicarslan S. (2026). Integrative Bioinformatics and Machine Learning Analysis Identifies Novel Molecular Biomarkers in Prostate Adenocarcinoma. Int. J. Mol. Sci..

[10] Kilicarslan S., Cicekliyurt M.M., Kilicarslan S., Hassan D.S.M., Samee N.A. (2026). Bioinformatics and machine learning integration reveals a novel 4-gene (GFUS, ARHGAP8, NBL1, and ACTB) biomarker model for prostate cancer. Discov. Oncol..

[11] Hosono N. (2019). Genetic defects of chromosome 5q and 7q in myeloid neoplasms. Rinsho Ketsueki.

[12] Hosono N. (2019). Genetic abnormalities and pathophysiology of MDS. Int. J. Clin. Oncol..

[13] Daniels N.J., Hershberger C.E., Gu X., Schueger C., DiPasquale W.M., Brick J., Saunthararajah Y., Maciejewski J.P., Padgett R.A. (2021). Functional analyses of human LUC7-like proteins involved in splicing regulation and myeloid neoplasms. Cell Rep..

[14] Kenny C.J., McGurk M.P., Schuler S., Cordero A., Laubinger S., Burge C.B. (2025). LUC7 proteins define two major classes of 5′ splice sites in animals and plants. Nat. Commun..

[15] Jourdain A.A., Begg B.E., Mick E., Shah H., Calvo S.E., Skinner O.S., Sharma R., Blue S.M., Yeo G.W., Burge C.B. (2021). Loss of LUC7L2 and U1 snRNP subunits shifts energy metabolism from glycolysis to OXPHOS. Mol. Cell.

[16] Li C., Feng L., Luo W.W., Lei C.Q., Li M., Shu H.B. (2021). The RNA-binding protein LUC7L2 mediates MITA/STING intron retention to negatively regulate innate antiviral response. Cell Discov..

[17] Shen L., Li C., Chen F., Shen L., Li Z., Li N. (2021). CRISPR/Cas9 genome-wide screening identifies LUC7L2 that promotes radioresistance via autophagy in nasopharyngeal carcinoma cells. Cell Death Discov..

[18] Liu X., Xie S., Jiang X., Song S., Wang L., Li S., Lu D. (2024). LUC7L2 accelerates the growth of liver cancer cells by enhancing DNA damage repair via RRAS. Cells Dev..

[19] Yue Q., Wang Z., Shen Y., Lan Y., Zhong X., Luo X., Yang T., Zhang M., Zuo B., Zeng T. (2024). Histone H3K9 Lactylation Confers Temozolomide Resistance in Glioblastoma via LUC7L2-Mediated MLH1 Intron Retention. Adv. Sci..

[20] Vaicekauskaite I., Juodakis J., Kazlauskaite P., Ciurliene R., Smailyte G., Lazutka J.R., Sabaliauskaite R. (2026). Development and validation of gene expression-based signature for high-grade serous ovarian cancer. J. Ovarian Res..

[21] Sharifnia T., Wawer M.J., Goodale A., Lee Y., Kazachkova M., Dempster J.M., Muller S., Levy J., Freed D.M., Sommer J. (2023). Mapping the landscape of genetic dependencies in chordoma. Nat. Commun..

[22] Lai J., Yang H., Xu T. (2021). Systemic characterization of alternative splicing related to prognosis and immune infiltration in malignant mesothelioma. BMC Cancer.

[23] Vandell A.G., McDonough C.W., Gong Y., Langaee T.Y., Lucas A.M., Chapman A.B., Gums J.G., Beitelshees A.L., Bailey K.R., Johnson R.J. (2014). Hydrochlorothiazide-induced hyperuricaemia in the pharmacogenomic evaluation of antihypertensive responses study. J. Intern. Med..

[24] Alghamdi M.A., Al-Eitan L., Ibdah R., Bani Khalid I., Darabseh S., Alasmar M., Ataa A. (2025). Genomic Insights into Blood Pressure Regulation: Exploring Ion Channel and Transporter Gene Variations in Jordanian Hypertensive Individuals. Medicina.

[25] Yang Y., Ai Z., Liu D., Gao X. (2025). Inhibition of lactylation by LRP1 expression increases the risk of intervertebral disc degeneration: A multi-omics summary-based Mendelian randomization analysis. Medicine.

[26] AlShail E., Alahmari A.N., Dababo A.A.M., Alsagob M., Al-Hindi H., Khalil H., Al Masseri Z., AlSalamah R., Almohseny E., Alduhaish A. (2023). A molecular study of pediatric pilomyxoid and pilocytic astrocytomas: Genome-wide copy number screening, retrospective analysis of clinicopathological features and long-term clinical outcome. Front. Oncol..

[27] Yao S., Yuan C., Shi Y., Qi Y., Sridha R., Dai M., Cai H. (2022). Alternative Splicing: A New Therapeutic Target for Ovarian Cancer. Technol. Cancer Res. Treat..

