# Supplementary material for: Pan-Cancer Analysis Identifies LUC7L2 as a Prognostic Biomarker and Potential Functional Regulator of Prostate Cancer Progression
Source: Biomedicines. 2026 Oct 9;14(10):2294. doi: 10.3390/biomedicines14102294 (PMC13702000; doi:10.3390/biomedicines14102294)
Supplement: Supplementary file 1 [file biomedicines-14-02294-s001.zip › biomedicines-4547971-supplementary.pdf]

Supplementary Table S1. Small interfering RNA (siRNA) sequences used in this study.

siRNA	Target gene	Sense strand (5′–3′)	Antisense strand (5′–3′)
si-LUC7L2-1	hsa-LUC7L2 (ID:51631)	GAACGUGUUCAUGAGUUAAAUTT	AUUUAACUCAUGAACACGUUCTT
si-LUC7L2-2	hsa-LUC7L2 (ID:51631)	GAAGUCUGCUCUGCCUAUUUATT	UAAAUAGGCAGAGCAGACUUCTT
si-LUC7L2-3	hsa-LUC7L2 (ID:51631)	GAGAGAAGCUUGAAGAAUUAATT	UUAAUUCUUCAAGCUUCUCUCTT
si-NC (negative control)	—	UUCUCCGAACGUGUCACGUTT	ACGUGACACGUUCGGAGAATT

The two terminal dT nucleotides (dTdT) are 3′ overhangs. si-NC denotes the non-targeting negative control siRNA. All siRNAs were synthesized commercially.
